# Safety of Yam-Derived (*Dioscorea* *rotundata*) Foodstuffs—Chips, Flakes and Flour: Effect of Processing and Post-Processing Conditions

**DOI:** 10.3390/foods8010012

**Published:** 2019-01-03

**Authors:** Celestina Omohimi, Clara Piccirillo, Vincenza Ferraro, Mariana C. Roriz, Mobolaji A. Omemu, Sandra M. Dias Santos, Sandrine Da Ressurreição, Louise Abayomi, Abdulraqaz Adebowale, Marta W. Vasconcelos, Oluwasegun Obadina, Lateef Sanni, Maria M. E. Pintado

**Affiliations:** 1 College of Food Science and Human Ecology, Federal University of Agriculture, Abeokuta, P.M.B. 2240, Ogun State, Nigeria; get2tina2@yahoo.com (C.O.); omemum@unaab.edu.ng (M.A.O.); rasaq.debo@gmail.com (A.A.); obadinaw@gmail.com (O.O.); sannilateef5@gmail.com (L.S.); 2 Universidade Catolica Portuguesa, CBQF—Centro de Biotecnologia e Quimica Fina—Laboratorio Associado, Escola Superior de Biotecnologia, 4200-375 Porto, Portugal; ferraro.vincenza@hotmail.com (V.F.); marianarorizcosta@gmail.com (M.C.R.); mvasconcelos@porto.ucp.pt (M.W.V.); mpintado@porto.ucp.pt (M.M.E.P.); 3 Polytechnic Institute of Coimbra, ESAC—Escola Superior Agraria de Coimbra, 3040-316 Coimbra, Portugal; sds@ecas.pt (S.M.D.S.); sandrine@ecas.pt (S.D.R.); 4 Natural Resource Institute, University of Greenwich, Medway Campus, Chatham Maritime, Kent ME4 4TB, UK; L.Abayomi@greenwich.ac.uk

**Keywords:** yam, processing, post-processing, bacterial contamination, aflatoxin, heavy metals, pesticide

## Abstract

The production of yam-derived (*Dioscorea* *rotundata*) foodstuffs is mainly performed by small and medium scale processors that employ old traditional methods. This can lead to differences in quality from processor to processor, and from location to location, with consequent safety concerns. As such, the effects of processing and post-processing phases (i.e., storage, transport, etc.) on the safety of some yam-derived foodstuffs—namely chips, flakes, and flour—has been evaluated, with a focus on bacterial and fungal contamination, aflatoxins, pesticides, and heavy metals (Pb, Ni, Cd and Hg). Yams harvested and processed in Nigeria were screened, being that the country is the largest producer of the tuber, with 70–75% of the world production. Results highlighted no presence of pesticides, however, many samples showed high levels of bacterial and fungal contamination, together with heavy metal concentrations above the recommended safety levels. No trend was observed between the items considered; it was noticed, however, that samples purchased from the markets showed higher contamination levels than those freshly produced, especially regarding bacterial and aflatoxins presence. The processing stage was identified as the most critical, especially drying. Nonetheless, post-processing steps such as storage and handling at the point of sale also contributed for chemical contamination, such as aflatoxin and heavy metals. The results suggested that both the processing and post-processing phases have an impact on the safety of yam chips, flakes, and flour.

## 1. Introduction

Yams (*Dioscorea* spp.) are important products in many countries. They are the third most consumed crops in the Sub-Saharan region, especially in West Africa [1,2,3], and are also largely consumed in the South Americas, India, and South-East Asia [4,5]. Yams represented a guarantee of food security for centuries, especially before the introduction of crops such as corn and maize [5,6]. 

The production of yams at a large scale is strongly linked to storage conditions. Fresh tubers, in fact, are very perishable due to microbe-induced rotting [7,8,9]. According to Girardin [10] and Ferraro et al. [5], the storage phase generally results in high losses, which can rise up to 25% of the raw weight. Physiological activities such as sprouting, transpiration, and respiration depend on the storage environment, mainly temperature and relative humidity [7,11]. To minimize losses, freshly harvested yams are processed into dry products to reduce the water activity as much as possible; as such, chips and flakes are often obtained [1,12]. As reported by Omohimi et al. [13], dried yam chips are produced through peeling, slicing, blanching, steeping, and sun-drying. Yam flakes are obtained with the same process as yam chips, where the drying process is faster due to the smaller size of the flakes. Yam chips and flakes can be further milled into flour, which can be reconstituted in boiling water to form a thick paste, consumed for meals in tropical areas as a source of carbohydrates [14,15]. 

The nutritional value of yam chips, flakes, and flour has been recently assessed by Omohimi et al. [13]. Aside from carbohydrates, yam-derived foodstuffs are also a good source of minerals, such as Ca, Mg, P, and K, and oligominerals, such as Zn, Co, Mn, and Cu. In virtue of their nutritional properties, yam-derived foodstuffs could eventually represent an alternative as gluten-free commodities, either to face coeliac disease in Europe and international countries, to satisfy consumer’s preferences, or food industrial processes. For the sake of comparison, the largely used tapioca starch derived from cassava can be taken as an example of a gluten-free source of carbohydrate products coming from tropical countries [5]. 

Currently, yam chips, flakes, and flour are produced by small and medium scale processors (cottage and rural processors) that employ non-standardized methods [5,13,14]. This can lead to differences in the quality of the final products from processor to processor, and from location to location, with consequent safety concerns. These aspects are particularly relevant for all the food items produced in developing countries, as often the production processes involve the use of old, traditional methods and with poor awareness of safety [13]. Different contaminants can be present in yam products, such as heavy metals and organic pollutants [5,8,16], as well as microorganisms in the form of bacterial strains, fungi, or mycotoxins [17,18]. 

Contamination can take place through different channels. Raw food products can already contain certain contaminants arising from agricultural practices (soil, water, tools, etc.) [16,19], which can likely be present in the foodstuffs after processing. On the other hand, the post-processing steps can lead to additional contamination, since food items can be in contact with or develop other pollutants during transport and storage. For instance, it is very common to dry both the crops and the derived food items in open land fields next to busy roads [5,20], resulting in exposure to car gases. These drying conditions also expose foodstuffs to insects and fungal attack. 

Concerning pesticides, literature reports their use on yam-derived foodstuffs mainly during storage, in order to reduce tuber losses and consequently maximize income (i.e., Actellic, Phostoxin, or a mixture of Gamalin 20 or kerosene with water). Such pesticides, however, can pose health and environmental pollution risks when above the recommended safety levels [21,22]. Contamination with mycotoxins can also pose serious threats to consumers’ health, as these secondary fungal metabolites can cause sickness or death in humans and animals [23,24]. Among all, aflatoxins are the most toxic and carcinogenic [25]. They are found in many tropical and subtropical countries, where the warm, humid weather provides optimal conditions for the growth of aflatoxinogenic molds (24–35 °C and equilibrium relative humidity of above 70%). Some literature data reported the presence of aflatoxin in both cassava- and yam-derived food items, such as flours and chips [8,26,27,28]. These studies, however, did not clarify when the contamination occurred (i.e., either during the processing or the post-processing stage). 

Monitoring of all phases is then a crucial step to understand where the contamination originates from, in order to improve the safety and promote commercialization of the yam-derived commodities at a large and international scale. Hence, in this work, a comparative investigation on the safety level of different yam foodstuffs is reported. The aims of this study were:

- To assess whether possible contamination (bacterial and fungal, presence of aflatoxin, heavy metals, and pesticides) takes place during the processing or the post-processing stages;
- To determine if different food items presented different kinds or levels of contamination.

The results of this investigation can likely give useful indications about the safety of different crop-derived foodstuffs consumed in other developing countries.

## 2. Materials and Methods 

### 2.1. Sample Collection and Preparation

The food items tested—yam chips, flakes, and flour—were purchased from food processors and from local markets and supermarkets in southwest Nigeria, which is by far the world’s largest producer of yam, accounting for 70–75% of the world production [2,6]. 

**Figure 1:**
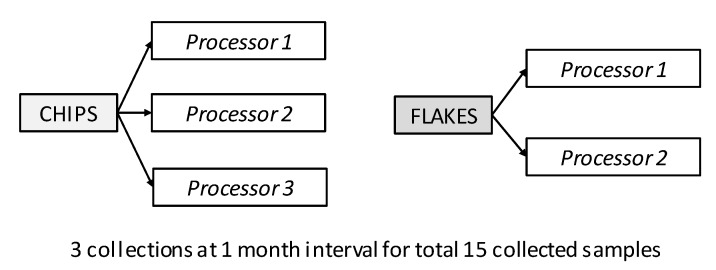
Scheme of collection of yam-derived foodstuffs from producers. The items collected were yam (*Dioscorea rotundata*) chips (three samples), and flakes (two samples). For all different products, collections at one month intervals were performed, for a total of 15 samples.

Samples were collected directly from producers and according to the scheme reported in Figure 1; yam chips were collected from three processors, while flakes were collected from two processors, and both in Saki, Oyo State. Initial sample size design for this research work was to collect samples from five yam chip and flake processors each. However, due to the constraint encountered in getting this number, three yam chip and two yam flake processors were chosen based on their willingness to participate in the study. The collection was performed during the processing season (between the months of November 2013 and March 2014). Samples were collected in three batches (A, B, and C) at one month intervals. A total of 15 samples were collected in all the batches. 

**Figure 2:**
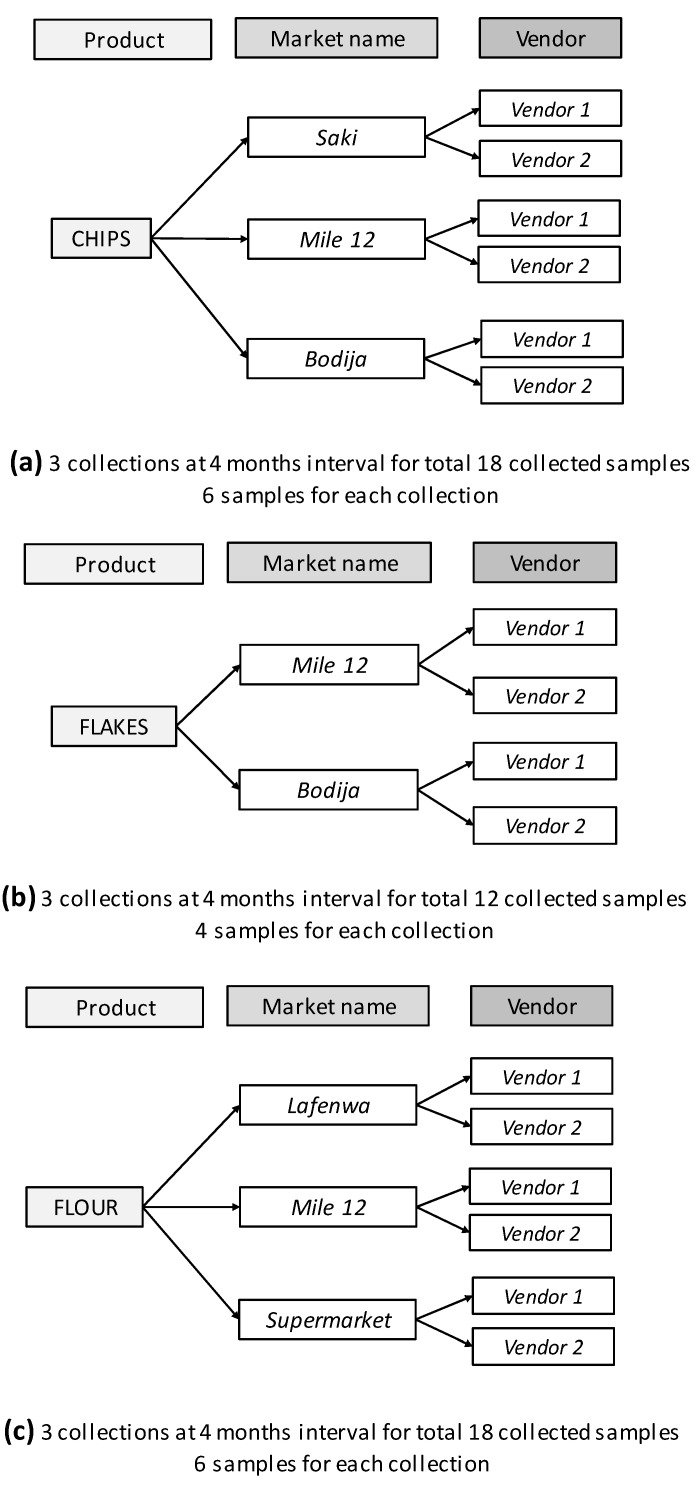
Scheme of collection of yam-derived foodstuffs from markets and supermarkets. The collected samples were yam (*Dioscorea rotundata*) chips (Figure 1a, six samples), flakes (Figure 1b, four samples), and flours (Figure 1c, six samples). For all different products, three collections at four month intervals were performed, for a total of 48 samples.

Figure 2, on the other hand, shows a schematic diagram of the samples collected from local markets and supermarkets. Dried yam chips were purchased from three markets—Saki in Oyo State, Bodija in Oyo State, and Mile12 in Lagos, southwestern Nigeria; yam flakes were obtained from two markets, Bodija in Oyo State and Mile12 in Lagos. Yam flour samples, on the other hand, were purchased from three sites—Mile12 market in Lagos, Lafenwa market in Abeokuta (Ogun State), and a supermarket in Lagos (packaged flours). In each market, samples were purchased from two different sellers; regarding the packed flours, on the other hand, two different brands were chosen. The collection was performed three times, at four month intervals; May 2013, September 2013, and January 2014 (batch A, B, and C, respectively). Sixteen samples were obtained from each collection, giving a total of 48 samples. 

In all cases, about two kilograms of the samples were collected, placed in sterile air tight bags, and transported to the laboratory. The yam chips and flakes were crushed and milled into flour using the laboratory attrition mill, sieved through 250 μm mesh, and stored in plastic containers at 4 °C for about 2 weeks until use. 

Each sample was analyzed to determine the moisture content, total bacteria count, *Staphylococcus aureus, Salmonella* spp., *Esherichia coli*, and total coliform counts, fungal counts and morphological identification, aflatoxins contamination, heavy metals, and pesticide residues, as detailed below. 

### 2.2. Moisture Content Determination

The moisture content was determined by oven heating 2 g of each sample at 105 °C up to a constant weight [29]. For each sample, three replicate tests were performed; the average value with the corresponding standard deviation was considered. 

### 2.3. Microbiological Analysis

#### 2.3.1. Isolation and Enumeration of Microorganisms 

The yam chips, flakes, and flour samples were tested to determine possible bacterial contamination using the spread plate method; samples were analyzed for total viable bacteria, *Staphyloccocus aureus*, *Salmonella* spp., total coliforms, *Esherichia coli*, and fungal count. All microbiological media used were prepared according to the manufacturer’s (OXOID, Thermo Scientific; Portugal) instruction. 

One gram (1 g) of representative samples was homogenized in 9 mL sterile peptone water (pH 7.0) by means of horizontal and vertical agitation for a few minutes to obtain a 1:10 dilution. A further ten-fold serial dilution was made up to 10^−6^ for colony count, and 0.1 mL each of appropriate dilutions was spread on 15–20 mL of the medium most appropriate for the growth of each strain. More specifically, Nutrient Agar, Mannitol Salt Agar, Salmonella Shigella Agar, MacConkey Agar, Methylene Blue Agar, and Sabouraud Dextrose Agar (SDA) were used for total viable bacteria, *Staphyloccocus aureus*, *Salmonella* spp., total coliforms, *Esherichia coli*, and fungal culture, respectively. For the fungi test, 0.05 mg of streptomycin sulphate was added to the SDA medium to suppress bacterial growth. 

Triplicates of each set up were made and the average value of the replicates was calculated, with standard deviation as associated error. All inoculated plates for bacteria tests were incubated at 37 °C for 24–48 h, while those for fungal detection were incubated at 26 ± 2 °C for 5–7 days. The colonies were counted and recorded. The number of colony forming units per gram (cfu/g) of the samples was calculated by multiplying the number of colonies by the dilution.

#### 2.3.2. Characterization of Mold Isolates 

Each of the fungi that emerged was sub-cultured onto a fresh SDA medium in a 15 mL slant bottle to obtain a pure culture. The cultural and morphological identification of the isolated molds were according to the mycelium structure, conditions of branches, presence of conidiospores, sclerotia, and shape [30,31]. The percentage frequency of occurrence of the mold isolate was determined using the equation below:

(1) $$Po=\frac{X}{N}\;\times \;100$$

where *Po* is the percentage of frequency of occurrence, *X* is the total number of individual mold isolate, and *N* is the total number of samples analyzed.

#### 2.3.3. Aflatoxin Determination

Aflatoxins B_1_, B_2_, G_1_, and G_2_ were analyzed by the chromatographic method reported by Ghali et al. [32], with some modifications. An amount of 25 g of homogenized sample was weighed into the blender, with the addition of 5 g of sodium chloride and 125 mL of extraction solvent (methanol/distilled water, 60:40 *v*/*v*); the mixture was then homogenized for 2 min by shaking vigorously. The mixture (*V*1) was then cleaned up as follows. First, *V*1 was filtered through a fluted filter paper. About 15 mL of the filtrate was pipetted afterward into a conical flask with glass stopper, and 30 mL of water was added and mixed. The diluted extract was filtered through a glass filter paper to give a clear filtrate (*V*2). From that second filtrate, 15 mL was passed through a C18 Sep-Pak separation column previously washed with a methanol/distilled water mixture (50:50 *v*/*v*). The eluate was then analyzed by reversed-phase high pressure liquid chromatography (RP-HPLC) with fluorescence detection and with the equipment Agilent 1260 Infinity (Agilent-Soquimica, Lisbon, Portugal). Mobile phase was distilled water/methanol/acetonitrile (60:20:20 *v*/*v*/*v*), at the flow rate of 1 mL/min. The column ZORBAX Eclipse XDB-C18, 4.6 mm × 25 cm, 3.5 μm (Agilent-Soquimica, Lisbon, Portugal) was kept at 40 °C all through the analysis. Post-column derivatization was done by bromination and before fluorescence detection at 365 nm excitation and 435 nm emission. Identification of each aflatoxin peak in the sample chromatogram was done by comparing the retention times with those of corresponding reference standards of aflatoxins B_1_, B_2_, G_1_, and G_2_ (Sigma-Aldrich, Sintra, Portugal), in the range of 0.1 to 10 μg/L.

### 2.4. Heavy Metals Analysis

Heavy metals were analyzed with the method reported in literature [33]. Prior to analysis, samples were dried to a constant weight, and underwent a microwave assisted digestion. For this, a weighed amount of each sample (200 mg) was mixed with 5 mL of 65% HNO_3_ in a Teflon reaction vessel and heated in a Speedwave^TM^ MWS–3+ (Berghof, Eningen, Germany) microwave system. The resulting digested clear solutions of the digestion procedure were transferred into 50 mL capacity tubes and then brought to 20 mL with deionized water. 

Presence and concentration of heavy metals (cadmium (Cd), lead (Pb), mercury (Hg), and nickel (Ni)) were determined using an inductively coupled plasma (ICP) optical emission spectrometer model Optima™ 7000 DV ICP-OES (Dual View, PerkinElmer Life and Analytical Sciences, Shelton, CT, USA) with radial plasma configuration. Standard plasma conditions were used, namely 1300 W for radio-frequency power, 1.5 mL/min pump rate, and 15.0, 0.2, and 0.8 L/min for plasma, auxiliary, and nebulizer gas flow, respectively.

### 2.5. Pesticide Residue Analysis

Pesticides were analyzed with the accredited method UNE-EN ISO/IEC 17025/2005 as reported by Camino-Sanchez et al. [34], with some modifications. An amount of 10 g of homogenous subsample of yam flour was weighed into a 50 mL centrifuge tube, to which 10 mL of acetonitrile was added; the mixture was shaken vigorously for 1 min. An amount of 4 g magnesium sulphate, 1 g dihydrate sodium citrate, and 0.5 g Na_2_H citrate sesquihydrate was added to the mixture in the same tube, shaken vigorously for 1 min, and then centrifuged for 5 min at 3000 rpm. Eight mL of extract was transferred into a centrifugation tube containing 25 mg. Primary secondary amine (PSA) and 150 mg MgSO_4_ was shaken manually for 30 min, after which it was centrifuged for 5 min at 3000 rpm. Afterwards, 5 mL of the upper layer was separated by a Pasteur pipette and transferred into a screw cup vial, and acidified with 10 µL 5% formic acid in acetonitrite (10 µL/mL extract). The cleaned and acidified extract was then transferred into auto-sampler vial and used for the multiresidue determination by gas chromatography coupled with tandem mass spectrometry (GC-MS/MS) by the equipment Varian CP-3800 coupled with a Varian 1200 quadrupole mass spectrometer (Varian, Soquimica, Lisbon, Portugal) and the capillary column Varian Factofour VF-5MS (30 m × 0.25 mm id × 0.25 µm film thickness). The standards used were Mix A and Mix B Supelco (Sigma-Aldrich, Portugal) for organochlorine pesticides and Mix A and Mix B of Restek (Pure chromatography, Restek Co., Bellefonte, PA, USA) for organophosphate pesticides; they included a total of 48 active compounds, such as carbofuran, chlorpyrifos, diazinon, dichlorvos, pirimiphos-methyl, dicofol, endrin, lindane, aldrin, dieldrin, and DDT. Calibration curves ranged from 0.006 to 0.1 mg/kg. 

### 2.6. Statistical Analysis

All determinations reported in this study were carried out in triplicate. In each case, a mean value and standard deviation were calculated. Analysis of variance (ANOVA) at 95% confidence level was also performed using the *SPSS* software, version 20.0 (IBM, Armonk, NY, USA). 

## 3. Results and Discussion

### 3.1. Sample Moisture Content

Table 1 and Table 2 show the moisture content level of the yam chips, flakes, and flour; it can be seen that values for the freshly produced samples (Table 1) and for those purchased from the markets (Table 2) are similar. In both cases, in fact, the majority of the samples showed a moisture level between about 8 and 13%. These data are in agreement with previous reports on similar yam-derived products [35]. Moisture is an important parameter which can affect the shelf-life and general acceptability of food; moisture levels above 13% in dried foods have been reported to promote spoilage and pathogenic microbial proliferation [36]. Considering the moisture content detected in markets and freshly processed samples, 12.5% of the markets and 27% of the freshly processed samples had moisture values above 13%. This could be attributed to either improper drying of the samples at the processing sites or moisture absorption during storage or at the point of sale. According to literature [37,38], producers use to stop the drying phase of chips after 3–6 days, leading to a moisture content around 20%, which strongly favors mold growth.

### 3.2. Microbiological Contamination of Yam, Chips, and Flour

**Figure 3:**
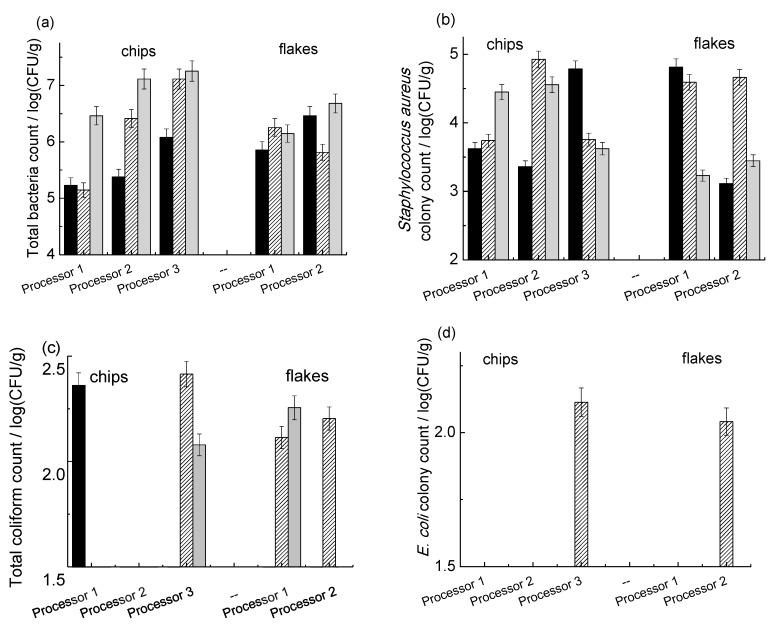

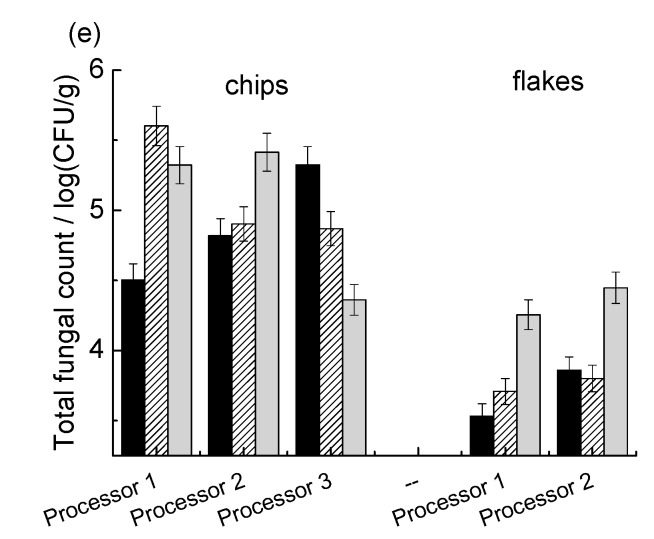
Bacterial counts for freshly processed chips, flakes, and flour yam samples. (**a**) Total bacterial count; (**b**) *Staphylococcus aureus*; (**c**) total coliform; (**d**) *Escherichia coli*; (**e**) total fungal counts. The black, patterned, and grey columns correspond to batches A, B, and C, respectively.

Figure 3 shows the microbial counts for the freshly produced chips and flakes. For the total bacteria (Figure 3a), about 60% of the samples present a count higher than 1 × 10^6^ cfu/g and higher than the limit set by the International Commission on Microbiological Specification for Food (ICMSF, 1998). Moreover, 3 (20%) of the 15 tested samples showed a bacteria population as high as 1 × 10^7^ cfu/g. It was observed that yam chip samples from batch C (January 2014, the last one collected) and from the three processors had higher levels of contamination than samples from batches A and B (November and December 2013, respectively). However, no particular trend was observed for the yam flake samples. Considering the *Staphylococcus aureus* presence (Figure 3b), this species was detected in all the samples; however, levels were never above 1 × 10^5^ cfu/g, which is considered the safety limit [39]. Total coliforms (Figure 3c) were detected in 40% of the samples, and in all cases, the colony count was always about 2 × 10^2^ cfu/g, well below ICMSF recommended limit (1 × 10^4^ cfu/g). For *Escherichia coli* (Figure 3d), on the other hand, only two samples in batch B showed the presence of this species. *Salmonella* species was not detected in any of the sample by direct plating. Regarding the fungi (Figure 3e), all samples showed contamination; the yam flakes were, overall, less contaminated than the chips, although the differences were not always statistically significant. 

**Figure 4:**
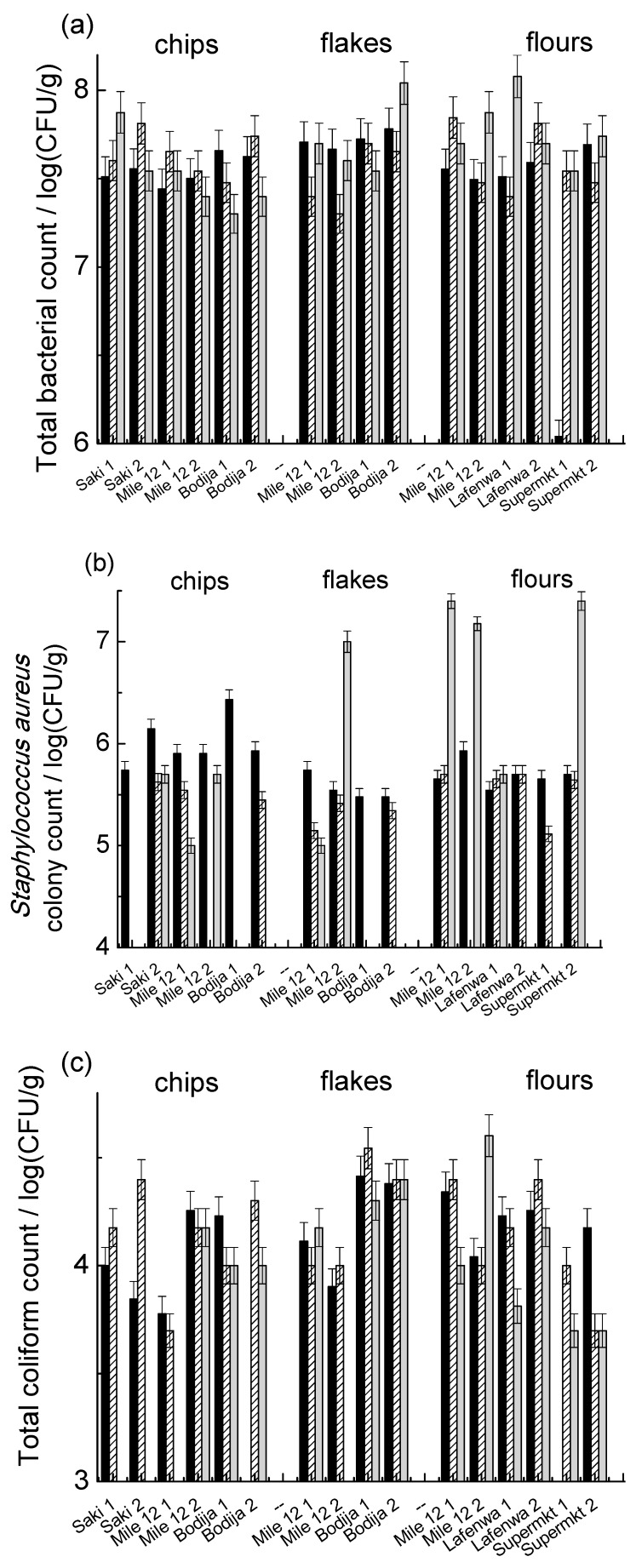

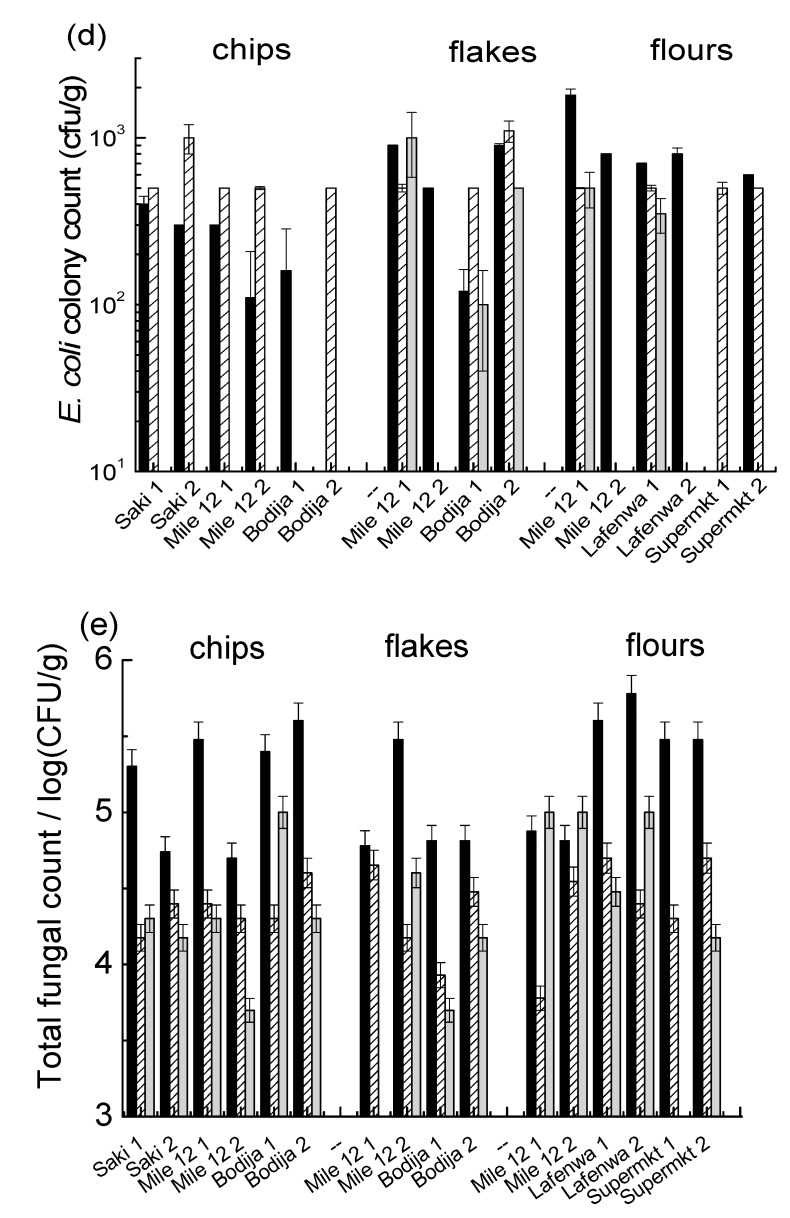
Bacterial counts for chips, flakes, and flour yam samples purchased in different markets. (**a**) Total bacterial count; (**b**) *Staphylococcus aureus*; (**c**) total coliform; (**d**) *Eshcerichia coli*; (**e**) total fungal counts. The black, patterned, and grey columns correspond to batches A, B, and C, respectively.

In Figure 4, the results for the food items purchased from selected markets are reported. For the total bacterial count (Figure 4a), a higher level of contamination than the freshly produced samples was observed, and in all the samples, the total count was above the set limit of 1 × 10^6^cfu/g. No particular trend was observed among the different sample sources or batches, and a yam flour sample in batch A from supermarket was the sole exception. Higher levels of contamination can also be seen for the *Staphylococcus aureus* presence (Figure 4b), where over 70% of the samples were contaminated. Overall, the highest contamination rate was observed in the batch C yam flour samples, while the lowest was in the batch B samples. For the total coliform count (Figure 4c), batch C yam flour was found to have the highest contamination, while yam chips showed the lowest contamination rate. Over 70% of batch A and B samples were contaminated with *Esherichia coli* (Figure 4d), while almost 70% of the batch C samples were free of that microorganism; in particular, none of batch C yam chip samples showed contamination. No *Salmonella* spp. were detected in the freshly processed samples by direct plating. Considering fungal growth (Figure 4e), fungi were detected in over 90% of the samples. Yam flakes showed the lowest contamination rate, while overall, batch A showed the highest (maximum count 6 × 10^5^ cfu/g). 

The high level of microbial contamination in both the freshly processed and market samples could be attributed to unhygienic handling of these commodities during processing and post-processing. Babajide et al. [14] reported on the microbial contamination of yam chips from some processing sites in southwest Nigeria, while in other studies a high microbial contamination of yam chip samples was observed from some selected markets in Togo [40] and southwest Nigeria [41]. Also, according to those previous studies, the high level of total bacterial count in all the market yam chip, flake, and flour samples, and in about 60% of the freshly processed yam chips and flakes, was above the limit set by the International Commission on Microbiological Specification for Food, which could be attributed to exposure of the samples to environmental conditions. These data showed that the sample form (chips, flakes, and flour) and the point of collection were determinant for the contamination. 

The commonly used drying conditions (such as rock surfaces, roadside, cemented floor, spreading on farmlands) could cause the commodities to be in contact with insects and toxigenic mycoflora from humans, environment, and soil; such mycoflora could then be inadvertently carried into storage. Mestres et al. [37] identified the drying stage in the production of yam chips as the critical control point (CCP). Somorin et al. [41] also identified the milling process of yam chips into flour as one of the means of microbial contamination. Other literature data also reported high levels of microbial contamination in yam chips from some selected processing sites (Oyo and Ogun states, Nigeria) [14]. 

The exposure of yam derived products without appropriate packaging in the markets for sale is another possible means of contamination [28]. Our study, however, shows that samples sold in supermarkets have comparable microbial contamination above the set limit, which might be due to microorganisms already present in the dried yam chips from which the yam flour was obtained or from the milling machine used [41]. The presence of *Staphylococcus aureus*, either in the freshly processed or market samples is indicative of human contamination, which could be from direct human contact, such as fingers, or indirectly through material used for processing [42]. The organism is a gram positive coccus that is resistant to heat, drying, and radiation, and associated with endotoxin characterized by a short incubation period (1–8 h), violent nausea, vomiting, and diarrhea. Detection of *Esherichia* *coli* in most of the samples assessed in this study suggests direct or indirect fecal contamination, because it is commonly used as a surrogate indicator.

The frequency of occurrence of mold isolates in batches A, B, and C market (yam chips, flakes, and flour) and freshly processed (yam chips and flakes) samples is presented on Table 3. Various mold species were isolated in the market samples, more specifically *Aspergillus flavus*, *Aspergillus niger, Aspergillus* spp., *Aspergillus fumigatus. Penicillium verricosum, Penicillium* spp., *Fusarium* spp., *Alternaria*, *Penicilum marneffei,* and *Mucor* spp. The most prevalent isolates are *Penicillium* spp. and *Aspergillus niger.* Table 3 also reports the frequency of mold isolates in batches A, B, and C of the freshly processed yam chip and flake samples; the same species detected in the market products were also detected in the fresh samples, with the exception of *Penicillium marneffei*, which was not detected. 

Differently to what observed for the bacterial contamination, the mold isolates data do not show significant differences between the freshly produced food items and those bought from market; this further signifies that the post-processing steps are not mainly responsible for this contamination. The mold isolates in this study are in line with the reports of some others in Nigeria, Benin Republic, and Togo [8,32,41,43,44]. As some molds have been identified as soil fungi, it could be deducted that the primary source of contamination is tuber contact with soil and absence of appropriate washing before processing; the use of bruised tubers or contact of healthy tubers with contaminated ones could also represent a critical factor [35]. 

According to Adegoke [45], the presence in food products of some of these molds, especially *Aspergillus flavus* and *Aspergillus niger*, is highly undesirable. Some of these molds, in fact, have been reported to have public health significance because of the production of mycotoxins, which have implication on consumers’ health and food shelf-life decreasing. This phenomenon is especially prevalent in developing countries, where *Aspergillus flavus* and *Aspergillus parasiticus* are mainly responsible for mycotoxin production [46].

### 3.3. Aflatoxin Contamination in Yam Chips, Flakes, and Flour

Considering aflatoxins, no contamination at all was found in all of the freshly processed yam chip and flake samples from the processors. For the foodstuffs purchased from the markets, however, aflatoxin B_1_, B_2_, G_1_, and G_2_ were detected in some samples (Table 4). Values obtained for Aflatoxin B_1_ ranged from 0.7 to 3.4 μg/kg, 0.5 to 3.4 μg/kg, and 0.3 to 0.8 μg/kg in batches A, B, and C, respectively. The results further show that 50% of the yam flour samples were contaminated with Aflatoxin B_1_, out of which 22% were above the set limit of 2 μg/kg for aflatoxin B_1_ in groundnuts, oil seeds, and other processed products intended for direct human consumption or use as an ingredient in foodstuffs [47]. Only 11% and 8% of the yam chips and flakes, respectively, were contaminated with the toxin but at levels below the set limit. For Aflatoxin B_2_, only flour samples from batches A and B were contaminated with values ranging from 0.1 μg/kg to 0.7 μg/kg, while no contamination was observed in yam chips and flakes. Considering the aflatoxin G_1_, only two chip samples and one flake sample showed contamination, corresponding to 11% and 8%, respectively. Flours, on the other hand, showed more contamination—aflatoxin G_1_ was detected in 8 out of 18 samples (45%), while aflatoxin G_2_ was also found in three flour samples. Aflatoxins G_1_ and G_2_ were found in neither chips or in flakes. 

Overall, results show higher levels of contamination in unpacked yam flour samples with respect to yam chips and flakes. Yam flour samples from the supermarket (i.e., the ones packed at the point of sale) did not contain toxins, except Batch A from Supermarket 1. Most of the contaminants were found in flour samples collected in batches A and B (November 2013 and December 2013, respectively) and this could be attributed to the sampling period during the rainy season, when the relative humidity (>70%) was high enough to permit or favor the proliferation of toxigenic fungi and the subsequent production of their secondary metabolites. This corroborates the findings of Makun et al. [43], who recorded higher mycotoxigenic fungal contamination during the rainy season than in the dry harmattan season among produce in Nigeria. The higher level of contamination in yam flour samples could also be attributed to the ability to absorb moisture from the environment due to the large surface area of flour particles. Nonetheless, a poor storage condition of the commodity by the sellers could have also had a role in contamination.

Regarding yam chips, results of this study differ from previous literature data for Nigeria [8,12,27,44,48] and Benin Republic [49,50], which reported high levels of Aflatoxin B_1_ contamination, above the 20 μg/kg total aflatoxin levels recommended by WHO and FAO (Food and Drug Administration of United States). The difference could likely be attributed to the different sampling locations and sampling periods. 

As stated above, the freshly processed yam chip and flake samples from the processors did not show any contamination with aflatoxins. These results may not seem to agree with the mold isolates data presented above; it has to be highlighted, however, that although *Aspergillus flavus* is a renowned aflatoxin producer, not all of its strains are actually capable of producing aflatoxin [50,51]. Indeed the interactions between some variables have to be taken into account; these include competing microflora in the samples for nutrients as well as unfavorable environmental conditions for toxin production. In addition, the presence of some active compounds added during parboiling of yam chips may also have an effect on the fungi growth rate, and subsequently, on mycotoxin production [52,35].

### 3.4. Heavy Metals Detection in Yam Chips, Flakes, and Flours

Concentration of the heavy metals lead, cadmium, and nickel in all the yam chip, flake, and flour samples, from the processing sites and from the selected markets, is reported in Table 5 and Table 6. 

Lead concentration was found to be from 0 to 0.42 mg/kg and 0 to 0.64 mg/kg for freshly produced yam chips and flakes, respectively; for the market samples, on the other hand, the ranges were from 0 to 1.02 mg/kg, 0 to 0.68 mg/kg, and 0 to 1.56 mg/kg for yam chips, flakes, and flour from the three batches, respectively. Overall, the market flakes were more contaminated than the freshly produced ones; in fact, 33% of the freshly processed flakes were found to be above the recommended limit of 0.2 mg/kg of Pb (as established for vegetables, cereals, and pulses) [53]. For the market flake samples, however, 58.3% had Pb concentration above the limit; the rate of highly contaminated food items, therefore, was significantly higher. This difference was not observed for yam chips, where comparable (and not significantly different) rates of samples with high Pb concentration was observed (33% versus 27.8%). Comparing all samples, it can be seen that the yam flour had the highest concentration of Pb contamination. This could be attributed to the ability of the yam flour samples to accumulate a higher amount of this metal from the environment due to the larger surface area, as compared with the yam chips and flakes. Considering just chips and flakes, however, flakes appear to have higher Pb content. This difference could be due to the fact that the processors dry the flakes by the road side where there is high vehicular movement. Yam chips, on the other hand, are dried on rocks or on farm lands. Indeed, some studies [54,55] have shown that exhaust from vehicles and gasoline combustion is one of the principal sources of Pb contamination in the environment. These results also show that the sellers or market and the period of collection were not determinant of the level of safety of the different commodities.

The Cd concentration in the market samples was observed to be in the ranges of 0.01–0.04 mg/kg, 0.02–0.12 mg/kg, and 0.01–0.11 mg/kg in yam chips, flakes and flour, respectively. For the freshly processed samples, however, Cd was not detected in any of the samples; for this metal, therefore, phases like storage and transport were the only sources of contamination. Cd can be present in air and soil (and in low amount in water) due to natural or anthropogenic activity. Volcanic eruptions, forest fires, rock weathering, and wind-blown dust are among the greatest natural sources of Cd. Nonetheless, the pollution caused by human activities can be crucial, with production of polyvinyl chloride plastic manufacturing, alloys, fungicides, solders, motor oil, and rubber and textile manufacturing being the main causes of Cd release in the environment [56]. The results of this study could be an indication that the contamination of the market samples with Cd could be as a result of accumulation in the air in the course of selling, as these commodities are usually exposed in the market places without packaging. However, none of the yam chip samples was above the recommended limit of 0.05 mg/kg Cd [57], while only 8.3% of the yam flakes and 11.1% of the flour were over this value. Lower levels of Cd have been observed in yam flour [58,59]; again, the variation could be attributed to difference in study location.

Regarding nickel, levels in the different commodities were found to be in the range of 0.07–0.45 mg/kg, 0.01–0.94 mg/kg, and 0.07–0.85 mg/kg in market samples of yam chips, flakes, and flour, respectively, while values for fresh yam chip and flake samples from processors ranged from 0.01 to 0.25 mg/kg and 0.3 to 0.27 mg/kg, respectively. It was observed that 96% and 80% of the market and processors’ samples, respectively, had level of nickel above the FAO and WHO tolerable limit of 0.05 mg/kg. Shin et al. [58] reported increased Ni contamination of commercial South Korean yam powder collected in South Korea after grinding. The higher level of contamination in market samples than the processors samples, especially in the yam flour, could be attributed to post-processing effects, such as milling, and during the selling phase where commodities are exposed to environmental pollution. High level of nickel contamination above the FAO and WHO tolerable limit in this study agrees with the report of Iweala et al. [59]. No mercury contamination was observed in this study in any of the commodities, irrespective of the sample collection point and batch. 

Overall, it could be concluded that some, although not all, of the studied food items showed contamination with heavy metals at worrying levels, above the recommended concentration. The effect of the post-processing treatment was significant, especially for metals such as Cd and Ni. Levels of heavy metal should be then taken into account before ingestion of the analyzed commodities owing to adverse effect on human health. Studies have shown that Pb affects practically the whole body; the adverse effects include reduction in intelligence quotient (IQ), increased blood pressure, and a range of behavioral and developmental defects [60]. Cadmium is a very toxic metal with no known biological function, and higher levels may cause health hazards [61]. According to Galadima et al. [62] and Luckett et al. [63], high levels of exposure to Cd is associated with irritation of the eyes and respiratory passage, damage to brain, liver, bones, and kidneys, and with association with some cancers, such as pancreatic cancer. Just like cadmium, nickel is also associated with damage to brain, liver, bones, and kidneys, and bronchitis, dermatitis, hypertension, rickets, and asthma at a high levels of exposure [62]. 

### 3.5. Pesticide Residues

No pesticide was detected in samples. This result could be attributed to the fact that the processors usually allow the products to stay for a minimum of three months in their storage facilities after the application of the pesticides and before taking the commodity to the market for sale. According to the processors, this duration of time is to allow the pesticide to evaporate in order to prevent some harmful effects when consumed immediately. Pesticide application is a post-processing operation practiced by the majority of the processors that do not intend to sell their commodities immediately after drying the yam chips, and in order to prevent insect attack and weevil infestation, which could reduce the market value of the products. These processors also tend to sell these commodities in time of scarcity (i.e., during the raining season, when more profits are made).

## 4. Conclusions

This study presented an investigation on several yam-derived foodstuffs (chips, flakes, and flours). Some products were acquired fresh at the processors’ sites, while others were purchased in markets and supermarkets. The following conclusions can be drawn:

- No significant difference was observed in the moisture levels between the freshly produced samples and the markets ones.
- Microbial contamination levels were observed in all samples, irrespective of the kind of food item or sources; overall, however, freshly processed foodstuff showed much lower contaminant concentrations (i.e., at least one order of magnitude lower for total bacterial counts).
- Similarly, heavy metal concentrations (Pd, Cd, and Ni) were higher in the market samples (especially the yam flour) than in those obtained from the processors.
- No aflatoxin was detected in the freshly produced foodstuffs, while higher levels were found in some market samples.
- No pesticide was detected in any samples.

The results of this study indicate that for microbial (bacterial and mold) contamination of the yam derived products, the processing stage is the most critical, especially drying. However, post-processing steps such as storage and handling at the point of sale were responsible for chemical contamination, such as aflatoxin and heavy metals. Environmental pollution (i.e., industrial or vehicular emissions) may also play a role.

Similar surveys should be performed with foodstuffs derived from other crops (like cassava), as well as with similar yam-derived food products obtained in different areas and conditions. This could help in understanding, and hence addressing, safety issues for food items produced from staple crops.

## Figures and Tables

**Table 1:** Moisture content level in batches A, B, and C of freshly processed yam (*Dioscorea rotundata*) chip, flake, and flour samples.

Sample	Batch A	Batch B	Batch C
**Yam Chips**			
Processor 1	12.67 ± 0.58 ^a, A^	13.33 ± 0.58 ^a, A^	11.67 ± 0.58 ^b, A^
Processor 2	14.33 ± 0.58 ^a, B^	10.33 ± 0.58 ^b, B^	12.33 ± 0.58 ^c, A^
Processor 3	12.67 ± 1.15 ^a, AB^	14 ± 0.0 ^a, A^	13.67 ± 0.58 ^a, B^
**Yam Flakes**			
Processor 1	10.33 ± 0.58 ^a, A^	8.33 ± 0.58 ^b, A^	10 ± 2.0 ^ab, A^
Processor 2	9.33 ± 0.58 ^a, A^	7.67 ± 0.58 ^b, A^	14.67 ± 2.31 ^c, B^

Values are mean ± standard deviations of three determinations. For each processor and for each product (chips, flakes, flour), different superscript minuscule letters among columns mean significant statistical differences (*p* ≤ 0.05) among batch A, B, and C. For each batch and for each product (chips, flakes, flour), different capital superscript letters among lines mean significant statistical differences (*p* ≤ 0.05) among processors. Batch A: November, 2013, Batch B: December, 2013, Batch C: January, 2014.

**Table 2:** Moisture content level in batches A, B, and C of yam (*Dioscorea rotundata*) chips, flakes, and flour samples from the market.

Sample	Batch A	Batch B	Batch C
**Yam Chips**			
*Saki 1*	10.00 ± 3.26 ^a, A^	12.87 ± 0.25 ^b, A^	8.67 ± 2.31 ^c, A^
*Saki 2*	11.00 ± 2.55 ^a, B^	11.81 ± 0.34 ^b, B^	8.0 ± 0.00 ^c, A^
*Mile12 1*	11.40 ± 0.28 ^a, B^	13.10 ± 0.34 ^a, C^	12.67 ± 1.16 ^a, B^
*Mile12 2*	12.10 ± 0.42 ^a, C^	12.38 ± 0.03 ^a, D^	12.00 ± 0.00 ^a, B^
*Bodija 1*	10.60 ± 0.28 ^a, D^	12.74 ± 0.36 ^b, D^	12.00 ± 0.00 ^c, B^
*Bodija 2*	10.60 ± 0.28 ^a, D^	12.01 ± 0.19 ^b, E^	10.67 ± 1.16 ^c, B^
**Yam Flakes**			
*Mile12 1*	11.80 ± 0.28 ^a, A^	11.84 ± 0.39 ^a, A^	9.33 ± 2.31 ^a, AB^
*Mile12 2*	10.00 ± 3.39 ^a, AB^	11.75 ± 0.14 ^a, A^	11.33 ± 1.16 ^a, B^
*Bodija 1*	13.20 ± 0.00 ^a, B^	13.09 ± 0.03 ^b, B^	10.00 ± 2.00 ^b, AB^
*Bodija 2*	15.40 ± 0.28 ^a, C^	12.13± 0.26 ^b, C^	7.33 ± 1.16 ^c, A^
**Yam Flour**			
*Mile12 1*	10.60 ± 3.11 ^a, A^	12.44± 0.15 ^b, A^	8.67 ± 1.16 ^b, A^
*Mile12 2*	10.40 ± 0.57 ^a, A^	11.26 ± 0.17 ^b, B^	9.33± 1.16 ^ac, A^
*Lafenwa 1*	10.20 ± 0.28 ^a, A^	14.61 ± 0.12 ^b, C^	10.67± 1.15 ^c, A^
*Lafenwa 2*	9.20 ± 0.00 ^a, B^	13.81 ± 0.18 ^b, D^	10.68 ± 1.17 ^c, AB^
*Supermarket 1*	10.00 ± 0.00 ^a, A^	10.03 ± 0.18 ^a, E^	12.67 ± 1.16 ^c, B^
*Supermarket 2*	10.00 ± 0.57 ^a, A^	9.66 ± 0.09 ^a, E^	12.00 ± 0.00 ^b, B^

Values are mean ± standard deviations of three determinations. For each processor and for each product (chips, flakes, flour), different superscript minuscule letters among columns mean significant statistical differences (*p* ≤ 0.05) among batch A, B, and C. For each batch and for each product (chips, flakes, flour), different capital superscript letters among lines mean significant statistical differences (*p* ≤ 0.05) among processors. Batch A: May, 2013. Batch B: September, 2013. Batch C: January, 2014.

**Table 3:** Frequency of occurrence of mold isolate in batches A, B, and C in market and freshly processed samples of yam (*Dioscorea rotundata*) chips, flakes, and flour.

Fungal Isolate	Frequency of Occurrence (%) in Market Samples (N = 16/batch)			Frequency of Occurrence (%) in Freshly Processed Samples (N = 5/batch)		
	Batch A	Batch B	Batch C	Batch A	Batch B	Batch C
*Aspergillus flavus*	12.5	-	62.5	40	20	-
*Aspergillus niger*	93.75	56.25	56.25	100	80	80
*Aspergilus* spp.	68.75	-	-	60	60	40
*Aspergillus fumigatus*	37.5	31.25	12.5	-	-	20
*Penicillium verricosum*	-	6.25	6.25	20	-	-
*Penicillium marneffei*	-	6.25	6.25	-	-	-
*Penicillium* spp.	87.5	25	18.75	60	20	20
*Mucor* spp.	-	25	25	-	-	20
*Fusarium* spp.	25	62.5	62.5	20	-	40
*Alternaria*	31.25	-	-	20	20	-

N = total number of samples analyzed for each batch. Note: - = not isolated.

**Table 4:** Aflatoxins levels (ppb) in market yam (*Dioscorea rotundata*) chips, flakes, and flour samples.

Sample	Batch A				Batch B				Batch C			
	B_1_	B_2_	G_1_	G_2_	B_1_	B_2_	G_1_	G_2_	B_1_	B_2_	G_1_	G_2_
**Yam Chips**												
*Saki 1*	-	-	-	-	-	-	-	-	-	-	-	-
*Saki 2*	-	-	-	-	-	-	-	-	-	-	-	-
*Mile12 1*	-	-	-	-	-	-	-	-	-	-	-	-
*Mile12 2*	-	-	-	-	-	-	-	-	-	-	-	-
*Bodija 1*	-	-	-	-	0.8	-	0.6	-	0.8	-	0.6	-
*Bodija 2*	-	-	-	-	-	-	-	-	-	-	-	-
**Yam Flakes**												
*Mile12 1*	-	-	-	-	-	-	-	-	-	-	-	-
*Mile12 2*	-	-	-	-	-	-	-	-	-	-	-	-
*Bodija 1*	-	-	-	-	1.2	-	1	-	-	-	-	-
*Bodija 2*	-	-	-	-	-	-	-	-	-	-	-	-
**Yam Flour**												
*Mile12 1*	1	-	1.8	0.1	1	-	1	-	-	-	-	-
*Mile12 2*	2.5	0.2	2.9	0.2	2.9	0.1	1.8	0.1	0.3	-	0.5	-
*Lafenwa 1*	0.7	-	0.7	-	3.4	0.7	-	-	-	-	-	-
*Lafenwa 2*	3.4	0.2	0.7	-	0.5	-	-	-	-	-	-	-
*Supermarket 1*	-	-	0.5	-	-	-	-	-	-	-	-	-
*Supermarket 2*	-	-	-	-	-	-	-	-	-	-	-	-

Note: - = below detection limit. Batch A: May 2013. Batch B: September 2013. Batch C: January 2014.

**Table 5:** Heavy metals concentration (mg/kg) in freshly processed yam (*Dioscorea rotundata*) chip and flake samples.

Sample	Batch A			Batch B			Batch C		
	Pb	Cd	Ni	Pb	Cd	Ni	Pb	Cd	Ni
**Yam Chips**									
Processor 1	-	-	0.12 *	-	-	0.11 *	0.42 *	-	0.01
Processor 2	0.23 *	-	-	0.07	-	0.25 *	-	-	0.16 *
Processor 3	0.35 *	-	0.06 *	-	-	0.14 *	-	-	0.02
**Yam Flakes**									
Processor 1	-	-	0.18 *	-	-	0.27 *	0.58 *	-	0.14 *
Processor 2	-	-	0.16 *	-	-	0.13 *	0.64 *	-	0.22 *

Note: - = below the detection limit. * = above the recommended limit (0.2 mg/kg for Pd and 0.05 mg/kg for Cd and Ni). Batch A: November 2013. Batch B: December 2013. Batch C: January 2014.

**Table 6:** Heavy metals concentration (mg/kg) in market yam (*Dioscorea rotundata*) chip, flake, and flour samples.

Sample	Batch A			Batch B			Batch C		
	Pb	Cd	Ni	Pb	Cd	Ni	Pb	Cd	Ni
**Yam Chips**									
*Saki 1*	-	0.03	0.19 *	0.70 *	0.03	0.23 *	-	-	0.12 *
*Saki 2*	1.02 *	0.01	0.22 *	0.88 *	0.03	0.18 *	-	-	0.08 *
*Mile12 1*	-	0.01	0.17 *	-	-	0.45 *	-	-	0.09 *
*Mile12 2*	-	0.04	0.30 *	-	-	0.14 *	0.27 *	-	0.07 *
*Bodija 1*	-	-	0.22 *	-	-	0.10 *	-	-	0.11 *
*Bodija 2*	-	0.03	0.27 *	0.06	-	0.32 *	0.55 *	-	0.20 *
**Yam Flakes**									
*Mile12 1*	-	-	0.27 *	0.62 *	-	0.41 *	0.37 *	-	0.01
*Mile12 2*	0.09	-	0.21 *	-	-	0.43 *	0.28 *	-	0.25 *
*Bodija 1*	0.32 *	0.05	0.94 *	0.17	-	0.77 *	0.06	-	0.06 *
*Bodija 2*	0.68 *	0.12*	0.33 *	0.24 *	0.02	0.35 *	0.24 *	-	0.08 *
**Yam Flour**									
*Mile12 1*	1.56 *	0.02	0.44 *	-	-	0.29 *	-	-	0.07 *
*Mile12 2*	-	0.11*	0.45 *	-	0.02	0.36 *	0.92 *	0.09 *	0.52 *
*Lafenwa 1*	0.57 *	0.01	0.13 *	0.45 *	0.04	0.85 *	0.73 *	-	0.13 *
*Lafenwa 2*	-	-	0.17 *	0.19	-	0.32 *	1.52 *	-	0.09 *
*Supermarket 1*	-	-	0.19 *	-	-	0.16 *	0.41 *	-	-
*Supermarket 2*	0.18	0.03	0.14 *	0.63 *	-	0.24 *	0.30 *	-	0.09 *

Note: - = below the detection limit, * = above the recommended limit (0.2 mg/kg for Pd and 0.05 mg/kg for Cd and Ni). Batch A: May 2013. Batch B: September 2013. Batch C: January 2014.
